# Trait sensitivity to negative feedback determines the effects of chronic stress and chronic mirtazapine treatment on anxiety and stress-coping strategies in rats

**DOI:** 10.1007/s00213-022-06273-8

**Published:** 2022-11-09

**Authors:** Paulina Surowka, Karolina Noworyta, Agata Cieslik, Rafal Rygula

**Affiliations:** Affective Cognitive Neuroscience Laboratory, Department of Pharmacology, Maj Institute of Pharmacology Polish Academy of Sciences, 12 Smetna Street, 31-343 Krakow, Poland

**Keywords:** Negative feedback sensitivity, Animal model, Stress, Mirtazapine, Rat

## Abstract

In this study, we examined whether trait sensitivity to negative feedback (NF) can interact with the effects of chronic stress and antidepressant treatment on anxiety and stress-induced coping strategies in rats. Results of the conducted experiments indicated that animals displaying trait insensitivity to NF were more prone to develop stress-induced anxiety than their NF-sensitive conspecifics. Moreover, an analysis of the behavioral patterns displayed by the NF-insensitive animals during the forced swim test (FST) revealed complementary (anxiety-driven) effects of trait sensitivity to NF on the strategy of coping with an acute, stressful situation. Finally, an analysis of the interactions between NF sensitivity and the effects of antidepressant drug — mirtazapine — revealed that in animals subjected to chronic stress, the effects of the drug on anxiety and coping strategies differ significantly between animals classified as NF insensitive and NF sensitive. The present results suggest that NF sensitivity screening could be potentially used to determine individual vulnerability to development of affective disorders and effectivity of their treatment.

## Introduction

Depression is one of the most debilitating mental illnesses (Smith 2014). Despite decades of research, the cognitive traits underlying this disorder still remain largely unknown. Several influential theoretical narratives (Corr 2004; Corr and McNaughton 2012; Kimbrel et al. 2007; Trew 2011) postulated that information processing biases could be considered a vulnerability factor for the etiology, maintenance, and recurrence of depression. This vulnerability has been conceptualized as a trait-like, latent, endogenous process, reactive to the effects of stress, which reside in genetic, biological, and psychological variables (Raedt and Koster 2010). However, to date, there have been very few studies that could directly support this theoretical claim. Implementation of sophisticated behavioral paradigms such as ambiguous-cue interpretation paradigm (Enkel et al. 2010) or probabilistic reversal learning (PRL) (Bari et al. 2010) used for the measurement of biases in information processing in experimental animals enabled the investigation of the abovementioned aspects of cognitive vulnerability to depression in animal models. In 2013, Rygula and colleagues demonstrated, for the first time, that in rats, similar to humans, cognitive judgment bias (optimism/pessimism) has both enduring trait and transient state components (Rygula et al. 2013). A trait represents a stable individual difference in the level of pessimism/optimism that is generally experienced, whereas a state captures the pessimism/optimism that may change based on the situation or contextual factors. They also showed that trait pessimism may determine the sensitivity of the animals to stress-induced anhedonia (Rygula et al. 2013). In 2019, Noworyta-Sokolowska and colleagues demonstrated a similar dichotomy concerning the levels of sensitivity to positive and negative feedback (NF), which were also postulated to be enduring and independent behavioral traits in rats (Noworyta-Sokolowska et al. 2019). In their follow up study, trait sensitivity to feedback was reported to interact with the effects of acute antidepressant treatment on anxiety and motivation in rats (Surowka et al. 2020).

The sensitivity to feedback can be effectively measured in humans and laboratory animals using the abovementioned, fully translational PRL paradigm (Drozd et al. 2019). PRL creates a unique opportunity to investigate the sensitivity to NF by analyzing the ability of subjects to ignore misleading information (Drozd et al. 2019). In this task, two (sometimes more) stimuli (e.g., patterns on a computer screen) are presented on each trial, and subjects, using trial-and-error feedback after each response, learn to select the stimulus that is usually correct (rewarded on a majority (e.g., 80%) or punished or unrewarded on a minority (e.g., 20%) of trials) and to avoid the one that usually is incorrect (punished or unrewarded on a majority (e.g., 80%) and rewarded on a minority (e.g., 20%) of trials). This rule intermittently reverses such that the stimulus that was usually rewarded becomes usually punished and vice versa. Consequently, responses must be adjusted in order to gain the reward and avoid punishment. For successful completion of the task, subjects have to learn to ignore infrequent and misleading NF that arises from the probabilistic nature of the discrimination. This “ability to ignore” indexes the sensitivity to NF (Drozd et al. 2019).

The present study has been devoted to the investigation of a hypothesis that the effects of chronic stress and antidepressant treatment on the behavioral correlates of depressive symptoms in rats may be influenced by trait sensitivity to NF. In other words, we wanted to test if NF sensitivity can determine the vulnerability of rats to chronic stress and response to antidepressant treatment.

To accomplish this goal, the animals were screened in a series of PRL tests, and in this way, each rat was classified into one of the 2 groups: NF insensitive or NF sensitive. Subsequently, the animals were subjected to chronic, lasting 4 weeks, restraint stress (verified animal model of depression (Rygula et al. 2013; Drozd et al. 2017)) and parallel chronic (2 weeks) treatment with antidepressant drug mirtazapine. Since the sensitivity to NF seems to theoretically interact with individual levels of anxiety and the ability to cope with stressful situations, the intergroup differences in the effects of stress and antidepressant treatment on anxiety-like behaviors and coping strategies were investigated using light/dark box (LDB) and forced swim (FST) tests.

## Materials and methods

### Ethic statement

Experimental protocols were reviewed and approved by the 2nd Local Institutional Animal Care and Use Committee at the Maj Institute of Pharmacology Polish Academy of Sciences in Krakow (Permission no. 242/2017).

### Subjects and housing

In the present study, we used 80 male *Sprague–Dawley* rats (Charles River, Germany) weighing 175–200 g (about 10 weeks old) upon arrival. Rats were kept in groups (four animals/cage) under controlled temperature (21 ± 1 °C) and humidity (40–50%) under a 12/12 h light/dark cycle (lights on at 7:00 h). The cage size was 56 (L) × 35 (W) × 21 (H) cm. The cages were enriched with wooden blocks and plastic pipes.

During the entire experiment, rats were mildly food restricted to 85% of their free-feeding weight (according to normal growth curve recommended by the laboratory rodent supplier—Charles River Research Models and Services Catalogue) by providing 15–20 g of food pellets per rat per day (standard laboratory chow). Food restriction began 1 week before behavioral training. Water was available ad libitum.

The experiments were performed during the light phase of the light/dark cycle.

### Apparatus

The PRL training and testing was performed in 16 computer-controlled operant conditioning boxes (Med Associates, St. Albans, Vermont, VT, USA). Boxes were equipped with a fan, light, speaker, a food dispenser set to deliver a sucrose pellet (Dustless Precision Pellets, 45 mg; Bio-Serv, Flemington, NJ, USA), and two retractable levers which were located on opposite sides of the feeder. We have programmed the experimental protocols using Med State notation code (Med Associates). The data were analyzed using a custom-written R program. The experimental procedure for the PRL task used in this study was a modified version of the procedures used and described previously by Bari and colleagues in 2010 (Bari et al. 2010) and has been described in detail elsewhere(Noworyta-Sokolowska et al. 2019; Rychlik et al. 2017).

### Measuring feedback sensitivity using the PRL test

#### Initial training

The initial instrumental training was already described elsewhere (Noworyta-Sokolowska et al. 2019; Rychlik et al. 2017). In the first stage, one of the levers (left/right counterbalanced between stages/animals) was extended, and every press on this lever was rewarded with sugar pellet delivery (fixed ratio schedule of reinforcement 1:1), after which the lever retracted for 3 s (inter-trial interval (ITI)) before the next trial commenced. No response within 10 s from lever presentation was marked as an omission, and a criterion of less than 20% omissions had to be met before progressing to the second stage of the training. Training sessions lasted 30 min, and there was no pre-set limit of trials. The second stage of training consisted of random presentations of either the left or right lever, each of which had to be pressed at least 30 times in 30 min. To avoid side bias during the PRL task, animals had to respond with similar frequency on both levers. This was achieved by training the rats to a criterion of less than 7.5% omissions on each lever (i.e., less than 15% total omissions but equally distributed between the levers) for 3 consecutive training days. After attaining this criterion, the animals were ready to be tested in the PRL procedure.

#### PRL training and testing

After the initial instrumental training, the rats were trained in the PRL paradigm. Each PRL training session consisted of 200 trials, and each trial lasted for a maximum of 22 s. The start of a trial was signaled by the house light, which remained on until the end of the trial. Two seconds after the trial had started, both levers were presented, and one of them was randomly assigned as the “correct” lever, which delivered a reward 80% of the times it was pressed. A press on the other lever—the “incorrect” lever—would result in a rewarding outcome only 20% of the times it was pressed. No response in 10 s triggered the 5-s intertrial interval (ITI) and was counted as an omission. The same ITI directly followed a punishing outcome, i.e., no reward on 20% of the correct and 80% of the incorrect lever presses. After every eight consecutive correct lever presses (regardless of the outcome), the criterion for the reversal of the outcome probabilities was reached. The previously correct lever now became incorrect and vice versa. This pattern was followed until the end of the session.

This training phase was repeated daily until the individual animals achieved sufficient performance levels. The criteria to be met were a minimum of 3 reversals completed during three consecutive training sessions, with less than 15% omissions per session.

#### Parameters measured in the PRL test

To monitor the sensitivity of rats to NF, the animals’ decisions were tracked on a trial-by-trial basis. To evaluate the sensitivity to NF, we assessed the ability of animals to ignore infrequent and misleading, punished (non-rewarded) outcomes on the “correct” lever. For this, the animal’s decisions to switch levers following such a misleading punishment (probabilistic lose-shifts) were scored and expressed as a ratio of all punished (unrewarded) outcomes on that lever.

#### NF sensitivity screening

The procedure used for NF sensitivity screening in the PRL was already described elsewhere (Noworyta-Sokolowska et al. 2019). In brief, after achieving a stable performance, animals that reached the criterion were subsequently tested in 10 consecutive PRL tests over 10 days. Based on this “sensitivity screening,” the rats were divided using a median split into sensitive and insensitive to NF. The division according to sensitivity to NF was made based on the average ratio of lever changes following misleading punishment (probabilistic lose-shifts) made by the animals across all 10 screening tests. Because the results of our previous studies have clearly indicated that such a dichotomous categorization based on median split is well suited to investigate NF sensitivity as a stable and enduring cognitive trait in rats (Noworyta-Sokolowska et al. 2019; Surowka et al. 2020; Noworyta and Rygula 2021; Rygula and Popik 2016), this way of data analysis has been extended over to the present research. The number of screening days following meeting the performance criterion was also based on the results of our previous experiments (Noworyta-Sokolowska et al. 2019; Surowka et al. 2020; Noworyta and Rygula 2021; Rygula and Popik 2016). To confirm the stability of the NF sensitivity traits, we additionally analyzed the “frequency of NF sensitivity,” expressed as the number of the PRL tests (out of the 10 comprising screening) in which an animal displayed sensitivity to NF, defined as a score above the median.

#### Chronic stress procedure

After classifying animals according to their sensitivity to NF, half of the rats were subjected to a chronic restraint stress procedure. The stress paradigm consisted of 1-h daily immobilization sessions that were performed over 4 consecutive weeks. Rats were transferred from a housing facility to the stress-room and then separated into the test room. The animals were placed into perforated, transparent plastic tubes (6.5 cm inner diameter) of an adjustable length. The restraint enabled normal breathing and limited movements of the head and limbs. Control animals were handled daily throughout the experiment.

#### LDB test

The procedure used for measuring anxiety in the LDB test was described in detail elsewhere (Noworyta-Sokolowska et al. 2019; Noworyta and Rygula 2021). In brief, the tests were performed in 4 computer-controlled Seamless Open Field Arenas for Rat (L 43.38 × W 43.38 × H 30.28 cm) equipped with 16 infrared emitters and photodetectors on each side of the box. Additionally, on 2 sides of each arena, there were rows of emitters and detectors enabling measurement of vertical activity (Med Associates; St Albans, Vermont, USA). For the LDB test, a dark insert was used, which divided the chamber into two equally sized compartments — a light one and a dark one with a hole in between that allowed rats to move freely between the compartments. The data were collected using Med State software (Activity monitor, Med Associates).

Before the tests, the rats were habituated to the experimental room for 30 min. Activity in the LDB was measured for 5 min. The test started by placing a rat in the center of the dark zone. An additional light source was placed above the light zone of the test chamber to make it more anxiogenic for a rat.

The rats’ movements were detected by beam breaks, and the whole system was computerized, which enabled reliable measurement of the latency to leave the safety of the dark zone and proportion of time spent in the anxiogenic-light zone.

#### FST

The FST followed the method described by Porsolt, 1978 (Porsolt et al. 1978). Twenty-four hours after the final stress session, the animals were individually placed into glass cylinders (35.5 cm height; 23 cm diameter) containing 25 cm of water at 23 °C. After 15 min, they were transferred to a 30 °C drying environment for 30 min (the pre-test). The animals were returned to the cylinder 24 h later for 5 min (the test), and this session was recorded with a video camera. Fresh water was used for each rat, and the cylinder was cleaned. Experiments were performed between 09:00 and 17:00. Each test session was scored by two observers unaware of the treatments applied. A time-sampling technique (Page et al. 1999) was employed to score, every 5 s, one of the following behaviors: (a) immobility, defined as the minimum movements done by animals to keep their nostril above the water (i.e., floating); (b) swimming, active motions made by animals that result in movements within the cylinder (i.e., moving around); (c) climbing, defined as strong movements executed with forepaws in and out of the water usually against the walls. Thus, in a 5-min test, a total of 60 counts, including immobility, swimming, and climbing, was obtained.

#### Experimental Design and Drugs

**Fig. 1:**
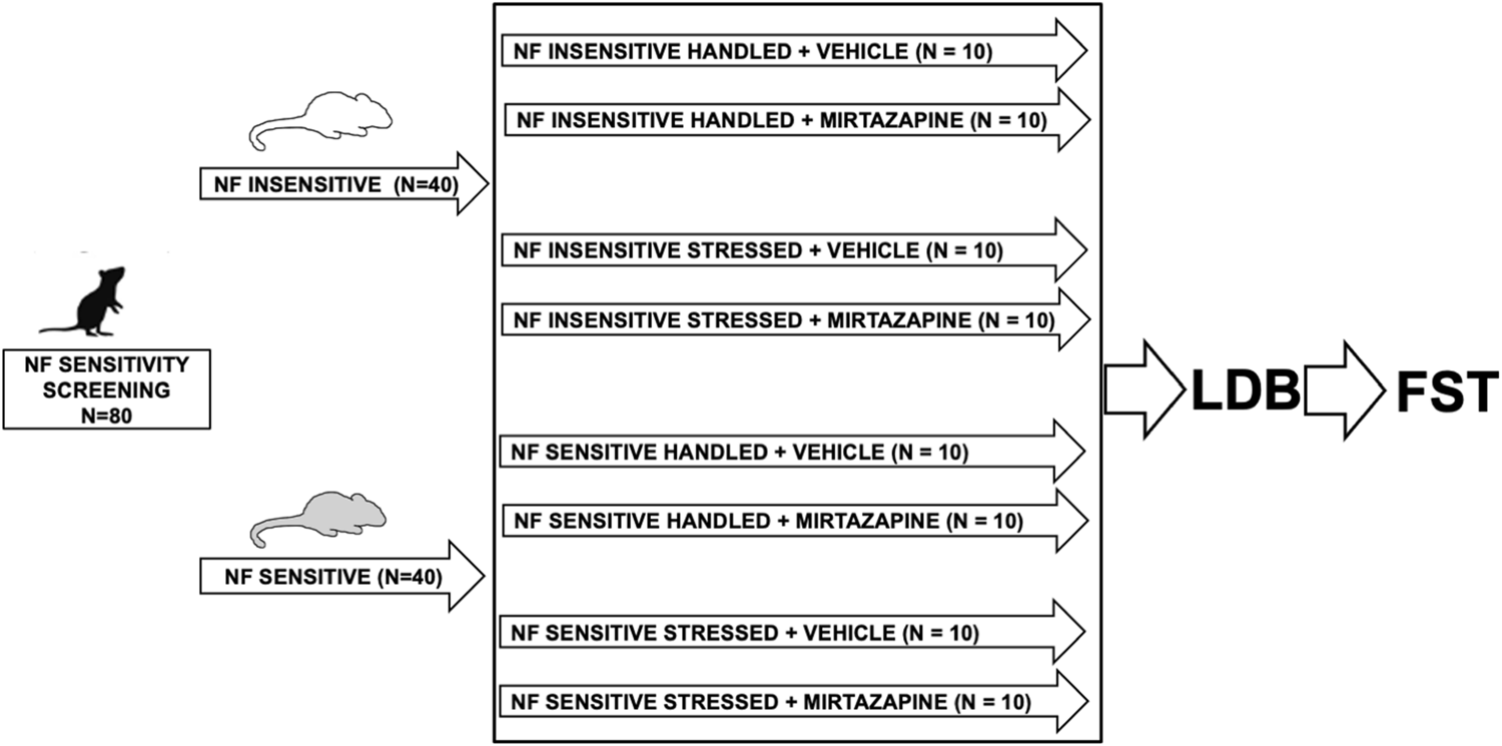
Experimental schedule. Abbreviations: negative feedback (NF), light dark box (LDB) and forced swim test (FST)

The experimental schedule is presented in Fig. 1. Initially, the rats were trained for the PRL test as described above. After achieving a stable performance, animals that reached the criterion were subjected to NF sensitivity screening, and based on its results, they were divided using a median split into sensitive and insensitive to NF groups. Subsequently, half of the NF insensitive (*N* = 20) and half of the NF sensitive (*N* = 20) animals were subjected to chronic restraint stress procedures lasting for 4 weeks (stressed groups). The other half of the NF insensitive (*N* = 20) and NF sensitive (*N* = 20) groups (control groups) were handled daily for the corresponding period of time. After 2 weeks of stress, half of the NF-sensitive, stressed animals (*N* = 10); half of the NF-sensitive handled animals (*N* = 10); half of the NF-insensitive, stressed animals (*N* = 10); and half of the NF-insensitive handled animals (*N* = 10) were subjected to 2 weeks of treatment with mirtazapine (mirtazapine-treated groups) applied along with the stress procedure. The numbers of animals in separate groups have been based on the sample sizes and power calculations estimated from our previous studies (see, e.g., (Noworyta and Rygula 2021)). Mirtazapine was purchased from TCI Europe (Zwijndrecht, Belgium). The drug (3 mg/kg, injected i.p., in a dose volume of 1 ml/kg) was dissolved in an equimolar solution of citric acid and injected once daily, for a period of 2 weeks. The other half of the animals received corresponding injections of the vehicle solution (vehicle-treated groups). The dose of mirtazapine has been chosen based on its effectivity in other animal studies (Drozd et al. 2019; Noworyta and Rygula 2021). On the last day of the stress and treatment regimes, the animals were tested in the LDB test, and 24 h later, they were subjected to FST pretest. The FST test was performed 24 h later.

#### Statistics

The data were analyzed using SPSS (version 25.0, SPSS Inc., Chicago, IL, USA). The distribution of the experimental data was tested using the Kolmogorov–Smirnov test. The effects of trait sensitivity to NF, chronic stress, and chronic mirtazapine treatment on parameters measured in the FST were investigated using three-way ANOVAs with the between-subject factors of sensitivity (two levels: NF insensitive and NF sensitive), stress (two levels: control and stress), and treatment (2 levels: vehicle and mirtazapine). Homogeneity of variance and sphericity of ANOVA were verified using Levene’s and Mauchly’s tests, respectively. For pairwise comparisons, the values were adjusted using the Sidak correction (Howell 1997). All of the tests of significance were performed at *α* = 0.05.

## Results

All animals fulfilled the training criteria and qualified for PRL screening. Two rats showing a significantly different pattern of behavior in the FST and in the LDB were excluded from analysis based on the Grubbs test for outliers.

### NF sensitivity screening

**Fig. 2:**
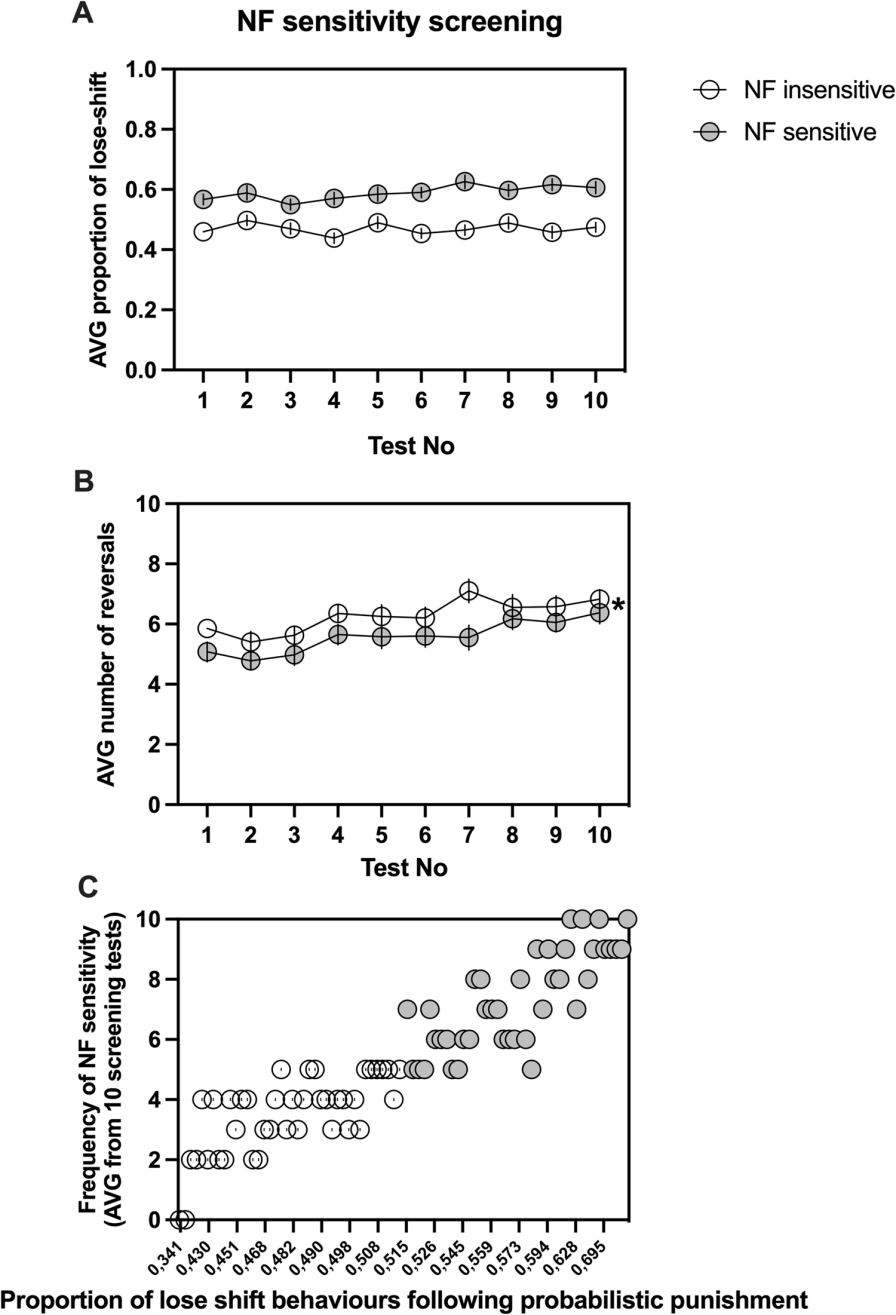
Results of the negative feedback (NF) sensitivity screening. **A** shows the average proportion of lose-shift behaviors following misleading punishment in rats classified as insensitive (open circles, *N* = 40) and sensitive (filled circles, *N* = 40) to NF across all 10 screening probabilistic reversal learning (PRL) tests. **B** shows the average number of reversals in rats classified as NF insensitive (open circles, *N* = 40) and NF sensitive (filled circles, *N* = 40) across all 10 screening PRL tests. **C** shows the average frequency of NF sensitivity in animals classified as NF insensitive (open circles, N = 40) and NF sensitive (filled circles, *N* = 40) during the NF sensitivity screening. The frequency is expressed as the number of PRL tests (out of the 10 comprising screening) in which an animal displayed the value of NF feedback sensitivity located above the median of the values from the entire cohort. Data are presented as the mean ± SEM. *p < 0.05 and ***p < 0.001 compared to the insensitive group

For the animals classified as NF insensitive, the average proportion of lose-shift behaviors following misleading NF ranged from 0.4386 to 0.4973 with an average 0.4696 ± 0.0184. For those classified as NF sensitive, the average proportion of probabilistic lose-shift behaviors ranged from 0.5498 to 0.6258 with an average of 0.5896 ± 0.0233. The sensitivity to NF in both subgroups was stable across the screening period (nonsignificant screening day × NF sensitivity interaction (*F*_(9,702)_ = 1.167, *p* = 0.3133, Fig. 2A)).

The average number of reversals made by the animals classified as NF insensitive during the screening period ranged from 5.4 to 7.1 with an average 6.27 ± 0.5301. For animals classified as NF sensitive, the average number of reversals ranged from 4.8 to 6.4 with an average of 5.580 ± 0.5252. The animals classified as NF sensitive made in average significantly less reversals than their NF-insensitive conspecifics (significant effect of NF sensitivity (*F*_(1,78)_ = 6.467, *p* = 0.013, Fig. 2B)). Reversal performance in both groups was stable (there was a nonsignificant interaction between screening day × NF sensitivity (*F*_(9,702)_ = 0.5565, *p* = 0.8330, Fig. 2B)).

The average frequency of NF sensitivity in animals classified as NF insensitive ranged from 0 to 5, with an average of 3.5 ± 1.3; in those classified as NF sensitive, the average frequency of NF sensitivity ranged from 5 to 10, with an average of 7.3 ± 1.4. The animals classified as NF insensitive were significantly less frequently sensitive to NF than the rats classified as NF-sensitive (*t* = 17.10, df = 39, *p* < 0.001, Fig. 2C).

### LDB

**Fig. 3:**
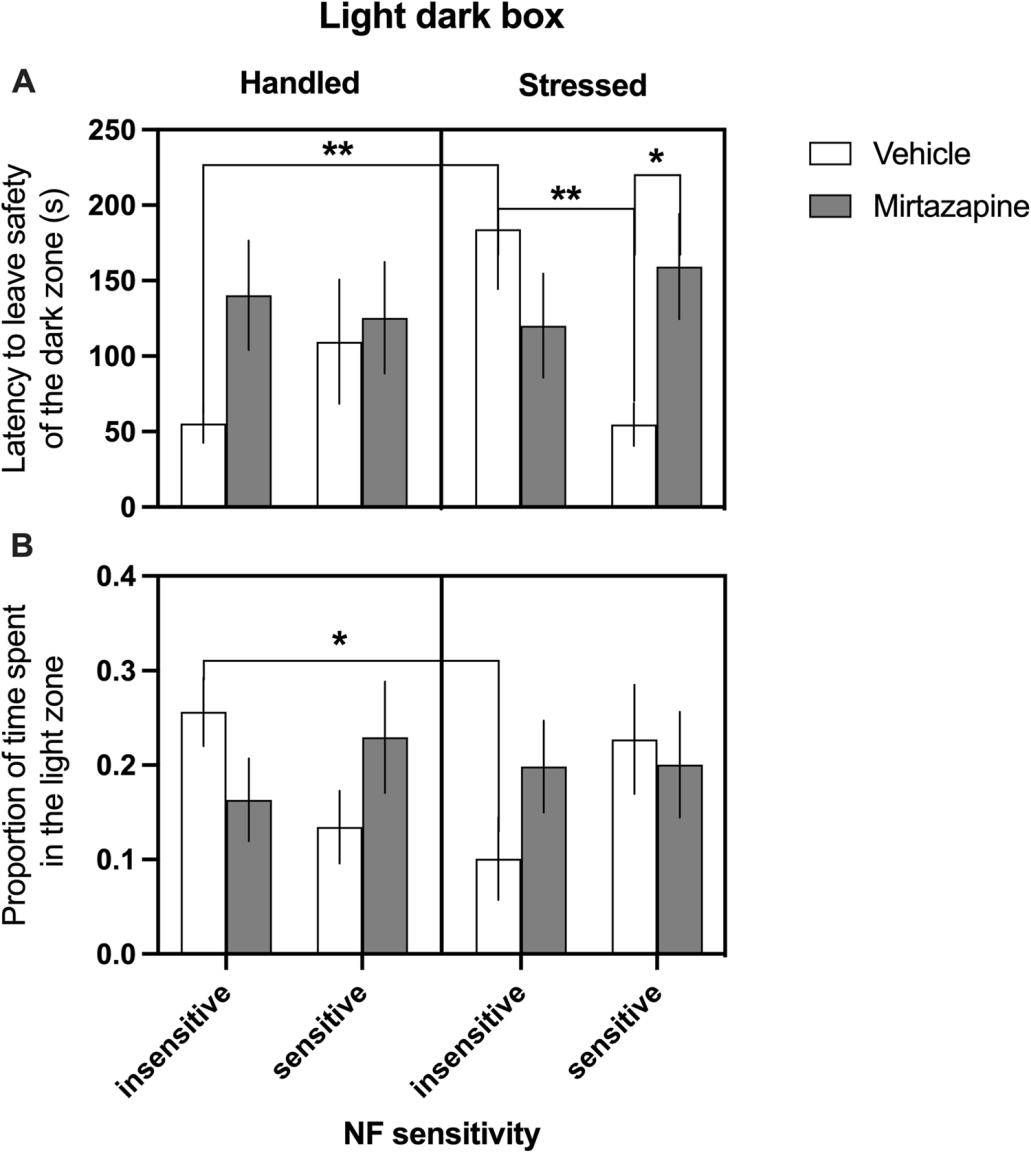
Effects of chronic stress and chronic administration of mirtazapine on anxiety-like behaviors in rats displaying trait insensitivity and trait sensitivity to negative feedback (NF). In the light dark box test, the effects of chronic stress on anxiety-like behaviors were most pronounced in rats classified as NF insensitive. This group reacted to the chronic stress with statistically significantly increased latency to leave the safety of the dark zone (**A**) and a significantly decreased proportion of time spent in the anxiogenic light zone (**B**) compared to their unstressed and their NF sensitive counterparts. Data are presented as the mean ± SEM (A and B). Panel A: * indicates a significant (p < 0.05) difference between NF insensitive, stressed, vehicle-treated, and NF insensitive, stressed, mirtazapine-treated groups of rats. **Significant (*p* < 0.01) difference between NF insensitive, handled, vehicle-treated, and NF insensitive, stressed, vehicle-treated as well as NF sensitive, stressed, vehicle-treated groups of rats. B: *Significant (*p* < 0.05) difference between NF insensitive, handled, vehicle-treated, and NF insensitive, stressed, vehicle-treated groups of rats

Analysis of the behavior of animals in the LDB revealed intergroup differences in the latency to leave the safety of the dark zone (significant NF sensitivity × stress × treatment interaction (*F*_(1,70)_ = 6.199, *p* = 0.015, Fig. 3A)). Post hoc analyses revealed that following chronic stress, the vehicle-treated animals belonging to NF insensitive group demonstrated significantly (*p* = 0.009) longer latency to leave the safety of the dark zone compared to their not stressed conspecifics (Fig. 3A). Moreover, following chronic stress, the animals classified as NF insensitive left the dark zone significantly (*p* = 0.01) later than their NF-sensitive conspecifics (Fig. 3A). These effects were not observed in mirtazapine-treated groups of animals. Following chronic stress, the NF-sensitive rats treated with mirtazapine left the safety of the dark zone significantly (*p* = 0.034) later than their vehicle-treated conspecifics (Fig. 3A). There were no statistically significant main effects of stress (*F*_(1,70)_ = 0.844, *p* = 0.361) or treatment (*F*_(1,70)_ = 2.196, *p* = 0.143).

Further analysis revealed also intergroup differences in the proportion of the time spent in the anxiogenic light zone (significant NF sensitivity × stress × treatment interaction (*F*_(1, 70)_ = 5,111, *p* = 0.027, Fig. 3B)). Post hoc analyses revealed that following chronic stress, the vehicle-treated animals belonging to NF-insensitive group spent significantly (*p* = 0.030) shorter time in the light zone than their handled conspecifics (Fig. 3B). There were no statistically significant main effects of stress (*F*_(1,70)_ = 0.167, *p* = 0.684) or treatment (*F*_(1,70)_ = 0.276, *p* = 0.601).

### FST

**Fig. 4:**
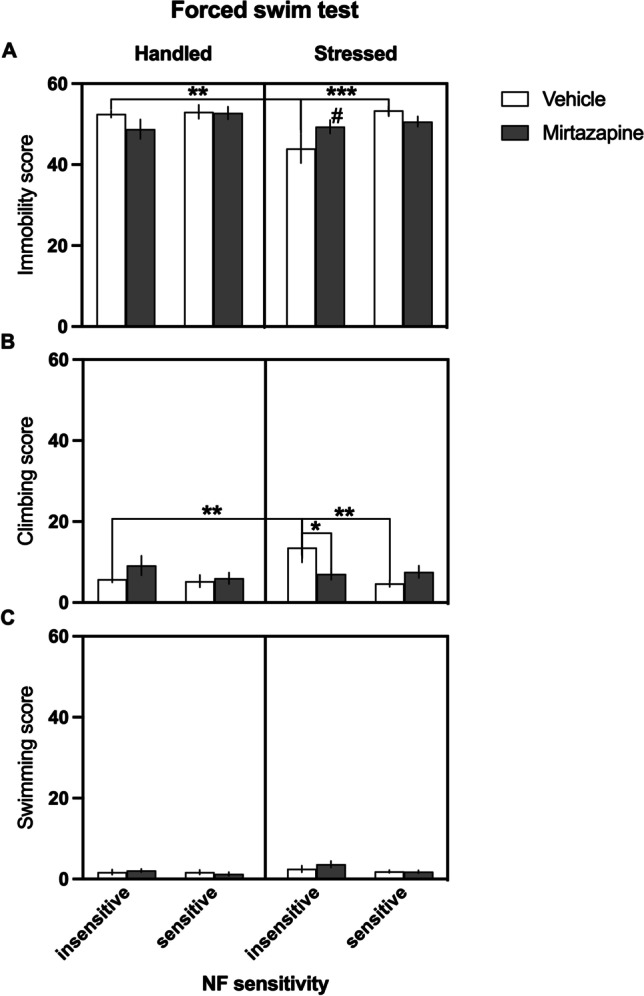
Effects of chronic stress and chronic administration of mirtazapine on coping strategies of rats displaying trait insensitivity and trait sensitivity to negative feedback (NF). Similar to light dark box, in the forced swim test, the effects of chronic stress on coping strategy were most pronounced in rats classified as NF insensitive. This phenotype reacted to the chronic stress with a statistically significantly lower score of immobility (**A**) and a significantly higher score of climbing (**B**) compared to their unstressed, as well as their NF-sensitive counterparts. There were no statistically significant intergroup differences in scores of swimming (**C**). Data are presented as the mean ± SEM. **A**: #trend (*p* = 0.056) towards statistically significant difference between NF insensitive, stressed, vehicle-treated, and NF insensitive, stressed, mirtazapine-treated groups of rats. **Significant (*p* < 0.01) difference between NF insensitive, handled, vehicle-treated, and NF insensitive, stressed, vehicle-treated groups of rats. ***Significant (*p* < 0.001) difference between NF-insensitive, stressed, vehicle-treated and their NF-sensitive, stressed, vehicle-treated counterparts. **B** *Significant (*p* < 0.05) difference between NF-insensitive, stressed, vehicle-treated and NF-insensitive, stressed, mirtazapine-treated groups of rats. **Significant (*p* < 0.01) difference between NF-insensitive, handled, vehicle-treated and NF-insensitive, stressed, vehicle-treated as well as NF sensitive, stressed, vehicle-treated groups of rats. #Statistical trend (*p* = 0.053) towards difference in the effects of mirtazapine treatment between NF-insensitive, stressed, vehicle-treated and NF-insensitive, stressed, mirtazapine-treated groups of rats

Analysis of the behavior of animals in the FST revealed intergroup differences in the immobility score (significant NF sensitivity × stress × treatment interaction (*F*_(1,70)_ = 4.238, *p* = 0.043, Fig. 4A)). Post hoc analyses revealed that following chronic stress, the vehicle-treated animals belonging to NF insensitive group demonstrated significantly (*p* = 0.004) lower immobility score compared to their not stressed conspecifics (Fig. 4A). Moreover, following chronic stress, the animals classified as NF insensitive, spent significantly shorter time immobile than their NF-sensitive conspecifics (*p* = 0.001, Fig. 4A). These effects were not observed in mirtazapine-treated groups of animals (Fig. 4A). There were no statistically significant main effects of stress (*F*_(1,70)_ = 2.976, *p* = 0.089) or treatment (*F*_(1,70)_ = 0.061, *p* = 0.806).

Further analysis revealed also intergroup differences in the time spent on climbing (significant NF sensitivity × stress × treatment interaction (*F*_(1, 70)_ = 4.742, *p* = 0.033, Fig. 3B)). Post hoc analyses revealed that following chronic stress, the vehicle-treated animals belonging to NF-insensitive group spent significantly (*p* = 0.002) longer time climbing compared to their not stressed conspecifics (Fig. 4B). Moreover, following chronic stress, the animals classified as NF insensitive spent significantly longer time climbing than their NF-sensitive conspecifics (*p* = 0.007, Fig. 4B). These effects were not observed in mirtazapine-treated groups of animals, although the drug significantly (*p* = 0.020) reduced climbing in NF-insensitive rats subjected to chronic stress, compared to their vehicle-treated conspecifics (Fig. 4B). There were no statistically significant main effects of stress (*F*_(1,70)_ = 1.463, *p* = 0.230) or treatment (*F*_(1,70)_ = 0.007, *p* = 0.935).

Analysis of the time spent on swimming revealed only a statistical trend (*F*_(1,70)_ = 3.883, *p* = 0.053), suggesting that the animals classified as NF insensitive spent more time on swimming than their NF-sensitive conspecifics. We observed no significant effects of the stress regime (*F*_(1,70)_ = 3.076, *p* = 0.084), or mirtazapine treatment (*F*_(1,70)_ = 0.276, *p* = 0.601) on this parameter (Fig. 4C). There were no statistically significant main effects of stress (*F*_(1,70)_ = 3.076, *p* = 0.084) or treatment (*F*_(1,70)_ = 0.276, *p* = 0.601).

## Discussion

The results obtained in the present experiments demonstrated that trait sensitivity to NF can interact with the vulnerability of animals to the effects of chronic stress. Following 4 weeks of stressful restraint sessions, the animals classified as insensitive to NF showed significantly longer latency to leave the safety of the dark zone and spent less time in the anxiogenic light zone of the LDB apparatus than their NF-sensitive and unstressed counterparts. These NF sensitivity–associated effects of stress on anxiety have been almost directly reflected in the coping strategies undertaken by NF-insensitive and -sensitive animals confronted with the acute stressor of the forced swimming. Following 4 weeks of the restraint stress, the rats classified as NF insensitive spent significantly less time immobile and significantly more time climbing the walls of the experimental cylinders than their sensitive and handled counterparts. Notably, an analysis of the interactions between trait sensitivity to NF and the effects of the antidepressant drug mirtazapine revealed that in animals subjected to chronic stress, the effects of antidepressant treatment differ significantly between the individuals classified as NF insensitive and NF sensitive.

A significant interaction between the trait sensitivity to feedback and anxiety has been previously demonstrated by Noworyta and Rygula 2021 in the context of acute antidepressant treatment (Noworyta and Rygula 2021). In the abovementioned study, the effects of acute administration of the selective serotonin reuptake inhibitor, escitalopram, on the anxiety of rats were determined by the level of sensitivity to positive and negative feedback. The present results confirmed and extended this observation. Significant differences in the effects of stress on the anxiety that were observed between the animals displaying trait sensitivity and insensitivity to NF, and reflected by the behavioral patterns recorded in the FST, unequivocally demonstrated the crucial role of NF sensitivity in coping with stress. Interestingly, although the FST was traditionally considered the gold standard for studying depression-like behaviors in rodents, recent theories have questioned the interpretation that immobility represents “despair” and that escape-directed behaviors such as climbing represent the absence of a depression-like phenotype. In 2016, De Kloet and Molendijk proposed that immobility in the FST is an adaptive learned response and reflects a switch from active to passive coping strategies (Kloet and Molendijk 2016), and a year later, Jeffrey Anyan and Shimon Amir hypothesized that escape-directed behaviors in this paradigm are driven by anxiety (Anyan and Amir 2018). The present results seem to support both of those theses, indicating at the same time a modulatory role of sensitivity to NF. It seems that following chronic restraint stress, the NF-insensitive rats, which were more anxious in the LDB, were just too afraid to stop swimming in the FST. Notably, this anxiogenic effect of stress was diminished by chronic treatment with the antidepressant drug — mirtazapine — which significantly shortened the duration of escape-directed behaviors (climbing). These at first sight confusing data form a matching puzzle, where NF sensitivity–dependent and stress-induced anxiety, observed in LDB, produces an anxiety-driven increase in escape behaviors (climbing) resulting in a decrease in immobility, which in turn can be abolished by the anxiolytic effects of chronic treatment with mirtazapine. Indeed, there is evidence for the effectiveness of mirtazapine in several anxiety disorders such as generalized anxiety disorder (Gambi et al. 2005), social anxiety disorder (Muehlbacher et al. 2005), panic disorder (Ribeiro et al. 2001), and post-traumatic stress disorder (Davidson et al. 2003). What is even more relevant to the present study, the drug has been found to relieve anxiety symptoms in patients with comorbid anxiety and depression (Goodnick et al. 1999). The anxiolytic profile of chronic mirtazapine treatment has been also observed in animal models like cocaine withdrawal–induced anxiety (Barbosa-Mendez et al. 2021), but to our knowledge, has never before been associated with a level of sensitivity to NF. Further studies should explain why the anxiogenic effects of mirtazapine on the latency to leave the safety of the dark zone observed in the NF-sensitive group were not observed in the FST. The most parsimonious explanation would imply that because this anxiogenic effect was observed only with the respect to the latency to leave the safety of the dark zone and not the proportion of the time spent in the light zone, it was partial and insufficient to modify the behavior of rats in the FST. In our opinion, this puzzling effect of mirtazapine observed in the NF-sensitive group of rats and absent in their NF-insensitive conspecifics adds to the growing body of evidence demonstrating that the effects of antidepressant treatment can be modulated by cognitive variables such as sensitivity to feedback (Noworyta and Rygula 2021).

There are also two other issues that need to be discussed. One of them is the statistically significant difference between the NF-sensitive and NF-insensitive groups of rats, in the latency to leave the safety of the dark zone, observed following chronic stress. However, because this parameter was statistically significantly changed, as compared to handled animals, only in the NF-insensitive group, one can assume that this effect was specific to this group only. The other regards the food restriction procedure applied in our study, which could have resulted in the social hierarchy’s dependent access to the food and by that confounded the investigated traits. This however seems unlikely as the food restriction procedure used in the present study is widely applied in studies using operant conditioning and has been validated in several previous studies focused on sensitivity to feedback (Noworyta-Sokolowska et al. 2019; Surowka et al. 2020; Drozd et al. 2019; Rychlik et al. 2017; Noworyta and Rygula 2021; Cieslik et al. 2022). Most importantly, however, the division of animals has been made based on their sensitivity to NF, which is based on their reaction to perceived failure (probabilistic lose-shift behavior in the PRL), and not based on their sensitivity to a reward. Hence, the mentioned potential differences in the amount of consumed food resulting from social hierarchy seem to not play a major role in the interpretation of results.

## Conclusion

Although the relationship between sensitivity to NF, depression, and effectiveness of antidepressant treatment has been widely postulated and brought about by influential cognitive theories, until now, there has been no systematic preclinical study aimed at investigating these interactions in an animal model. In the present paper, we provided the first experimental evidence that trait sensitivity to NF can determine the effects of chronic stress and the effects of chronic antidepressant treatment on anxiety-induced coping strategies of rats. And although the present results call for further investigation of the neurobiological mechanisms involved and determination of whether trait sensitivity to NF interacts with molecular and physiological correlates of chronic stress and antidepressant treatment, they also strongly suggest that NF sensitivity screening could be used to determine individual vulnerability to affective disorders and their treatment.
